# Effect of Age on the Immune and Visceral Organ Weights and Cecal Traits in Modern Broilers

**DOI:** 10.3390/ani11030845

**Published:** 2021-03-17

**Authors:** Yordan Martínez, Edison Altamirano, Victoria Ortega, Patricio Paz, Manuel Valdivié

**Affiliations:** 1 Poultry Research and Teaching Center, Agricultural Science and Production Department, Zamorano University, P.O. Box 93, Valle de Yeguare, San Antonio de Oriente, Francisco Morazan, Tegucigalpa 11101, Honduras; edison.altamirano.2019@alumni.zamorano.edu (E.A.); victoria.ortega.2019@alumni.zamorano.edu (V.O.); 2 Agricultural Science and Production Department, Zamorano University, P.O. Box 93, Valle de Yeguare, San Antonio de Oriente, Francisco Morazan, Tegucigalpa 11101, Honduras; ppaz@zamorano.edu; 3 National Center for Laboratory Animal Production, P.O. Box 6240, Santiago de las Vegas, Rancho Boyeros, La Habana, Cuba; mvaldivie@ica.co.cu

**Keywords:** age, digestive organ, cecal pH, cecal lactic acid bacteria, broiler

## Abstract

**Simple Summary:**

Currently, due to the high developments achieved in the poultry industry especially in genetics, management, nutrition, health, and animal welfare, modern broilers reach slaughter weight at an earlier age, which in turn has brought about notable changes in the morphophysiology of these birds. The following research proposes to determine the effect of age on visceral and immune organ weight, cecal pH, and cecal lactic acid bacteria in Ross 308^®^ broilers, up to 10 days old. It was concluded that the immune and visceral organs increase their absolute and relative weight according to age and on days 9 and 10 the highest growth rate of the organs was found, furthermore, the colonization of the cecal lactic acid bacteria is established before 10 days of life (as the most critical stage), although with variable changes for intestinal pH. The correlation showed, in addition, a significant association between the organs evaluated, as well as for the cecum relative weight and the cecal lactic bacteria count. These results could contribute to updating knowledge on immunological activity, cecal microbiology, and the functioning of the digestive system, as well as for the development of new nutritional requirements and the optimization of dietary formulations.

**Abstract:**

This study aimed to determine the effect of age on the immune and visceral organ weights and cecal traits in modern broilers. 200 male Ross^®^ 308 broilers were randomly selected, then 20 broilers were slaughtered every day (up to 10 days old) after six hours of fasting. All the organs measured had a progressive increase in absolute weight as the days progressed, apart from the spleen, which decreased its absolute weight on day 5, even though on day 10 it showed the highest values. Moreover, the small intestine relative weight increased from the fourth to the ninth day and was correlated (*p* ≤ 0.05) with the relative weight of the proventriculus, gizzard, small intestine, and cecum, although without statistical association with the of the heart. There was a correlation between the cecum relative weight and the cecal lactic acid bacteria, and between the primary lymphoid organs. The pH (from 5.74 to 7.40) and cecal lactic acid bacteria (from 6.11 to 8.79 log 10 CFU/g) changed according to the age of the broilers. The results could contribute to the understanding of the physiology and intestinal microbiology of the first 10 days old of modern broilers, which is crucial to improve the genetic expression of these animals.

## 1. Introduction

The poultry industry’s rapid growth and the advances in genetic selection, management, and nutrition mean that broilers reach physiological slaughter weight at a younger age [1]. The genetic selection process has focused much more on increasing muscle mass with feeding efficiency [2] and a lesser extent on improving the digestive and immune system to respond to the stress generated by rapid growth [3].

The first week after hatching represents 20% of the chicken’s productive life, which increases its body weight from two to three times with considerable changes in the gastrointestinal and immune organs [4]. According to Schmidt et al. [2], the digestive system has evolved gradually, which has improved the absorption of essential nutrients; thus, gastrointestinal development plays a vital role in the early stages of chick growth due to the degradation and absorption of nutrients for the maintenance and production. In hatching, the chicken’s digestive system is anatomically complete; however, its functioning is immature and during its growth, there are changes such as an increase in the length, height, and intestinal density of the villi. According to studies, the intestine also reaches its maximum relative weight between the fourth and eighth days after birth compared to the gizzard and pancreas that do not have a high growth rate [5].

After hatching, the gastrointestinal microflora of the chick has several microorganisms that are transported through the mucosa, which form cell layers that invade and colonize from the mouth to the cecum [5]. Among the main functions of the intestinal microflora are digestion, metabolism, pathogen exclusion, and immune stimulation [6]. The colonization of the small intestine is broader due to its neutral pH, with the cecum being the gastrointestinal segment with the highest number of microorganisms [5]. The cecum is responsible for the absorption of water, electrolytes, and the production of volatile fatty acids, thus this physiological process stimulates immune function, which is beneficial for the host’s intestinal health [7].

Lymphoid organs are indispensable for the bird’s performance during the first week of life. These immune organs develop in the embryonic phase, and their maturation takes place in the first week after hatching [8]. Maternal antibodies deposited in the egg constitute the immunity of the birds during the first seven days old [5]. The main lymphoid organs are the thymus, bursa of Fabricius, and spleen. The antibodies are dispersed throughout the body via the bloodstream and lymphatic circulation, in which migration occurs in a directed way according to the response in each case in pathogen type function [9].

Although several investigations have been carried out to understand the digestive physiology and gut microbiology of broilers [2,4,5,6]. To our knowledge, few current studies have considered the daily changes that occur in the visceral and immune organs, as well as the pH and the cecal lactic acid bacteria and their functional relationship between them in fast-growing broilers in the most critical productive stage (1–10 days). Thus, it is necessary to carry out new research that considers the growth rate of these organs and the cecal traits of the modern broilers, which could contribute to understanding the weight growth of these new genotypes at an early age, as well as for the development of possible genetic improvements and new nutritional requirements and dietary formulations. Therefore, our research was to determine the effect of age on the visceral and immune organ weights, cecal lactic acid bacteria, and cecal pH in Ross 308^®^ male broilers.

## 2. Materials and Methods 

All the procedures adopted in carrying out this experiment were approved by the Science and Agricultural Production Department at the Zamorano University, San Antonio de Oriente, Honduras, and conducted according to the Guidelines for Experimental Animals (Reference number: 19104 and 19081). 

### 2.1. Study Site

The study was carried at the Poultry Research and Training Center of the Zamorano University, located in Valle del Yegüare at km 32 of the Tegucigalpa-Danli highway, Honduras. The training center is 800 m above sea level, with an average temperature of 26 °C and an annual rainfall of 1100 mm.

### 2.2. Birds, Experimental Conditions, and Diets

For this study, 200 one-day-old male chickens of the Ross^®^ 308 genetic line were randomly allotted in 10 pens (20 chickens/pen), where each animal constituted an experimental unit. The pens were made with a deep wood chip bed. Feed (Mash form) and water were offered ad libitum in hopper feeders and nipple drinkers, respectively. The room temperature and ventilation were controlled using gas breeders, curtain handling, and fans. The barn was disinfected according to environmental quality standards of Poultry Research and Training Center Protocol, 24 h before the chicks entered the experimental area, this was disinfected with quaternary ammonium (5%). The diets were formulated according to the nutritional requirements of Ross^®^ 308 broilers (Table 1) [10].

### 2.3. Absolute and Relative Weight of the Digestive and Immune Organs and pH Cecal

For 10 days, 20 male chick/day with six hours of fasting were randomly selected and weighed individually before slaughter on a Navigator OHAUS model N38110 scale with a precision of ±1 g (OHAUS ^TM^, Parsippany, NJ, USA). Birds were euthanized by a veterinarian using the mechanical cervical dislocation method. The digestive viscera (liver, heart, and pancreas), immune organs (thymus, spleen, and bursa of Fabricius), and digestive organs (proventriculus, gizzard, small intestine, and cecum) extracted and weighed using a BLAZE BL model 100-01-BK scale with a precision ±0.01 g (Dalman Enterprises Ltd, Wycombe, Buckinghamshore, UK). Afterward, the relative weight of the organs was calculated considering the bodyweight at slaughter [11]. Following the slaughter, the pH in the right cecum of 20 birds/treatment [12] was determined using an Oakton^®^ model 700 digital pH meter (Oakton Instruments, Vermon Hills, IL, USA). Before testing, the potentiometer was calibrated with pH buffers at 1.68, 4.01, 7.00, 10.01, and 12.45 according to the manufacturer’s recommendations. 

### 2.4. Microbiological Analysis

Besides, the left cecum of five birds/treatment were randomly taken, and the mucosa was scraped with a scalpel for microbiological culture. Each sample’s cecal content was placed in a sterile tube; weight was recorded and diluted with Butterfield’s phosphate buffered dilution water to a 1:9 ratio (weight:volume). Diluted cecal contents were homogenized, and serial dilutions (1/10) were made from it until dilution 10^5. Aliquots of 0.1 mL of each dilution were spread plated on the surface of MRS agar (Neogen Acumedia, Lansing, MI, USA) supplemented with methylene blue (0.016 g/1000 mL) at 37 °C with a pH of 5.6 for 48 h in anaerobiosis (Gas Pak^TM^ BBL, Cockeysville, MD, USA). Counts of lactic acid bacteria were reported as Log 10 CFU/g by colonies’ morphology on MRS + MB agar. Gram stain and catalase activity were tested on each type of colony reported [12]. A Labomed model LX400 light microscope (Labomed Inc., Los Angeles, CA, USA) was used to morphologically characterize the bacterial colonies. The microbiological tests were performed in the Food Microbiology Laboratory of the Zamorano University.

### 2.5. Statistical Analysis

Also, the data were processed by analysis of variance (ANOVA) of one-way in a completely randomized design, before carrying out the analysis of variance we proceeded to verify the normality of the data using the Kolmogorov–Smirnov test and for the uniformity of the variance by Bartlett’s test, where necessary, Duncan’s test was used to determine the differences between means (*p* ≤ 0.05). Additionally, Pearson correlations were done to verify the association between the relative weights of the organs and the relationship between relative weight, pH, and cecal lactic acid bacteria. All the analyzes were made according to the statistical software SPSS version 23.0 (SPSS Inc., IBM Corporation, New York, NY, USA).

## 3. Results

**Figure 1:**
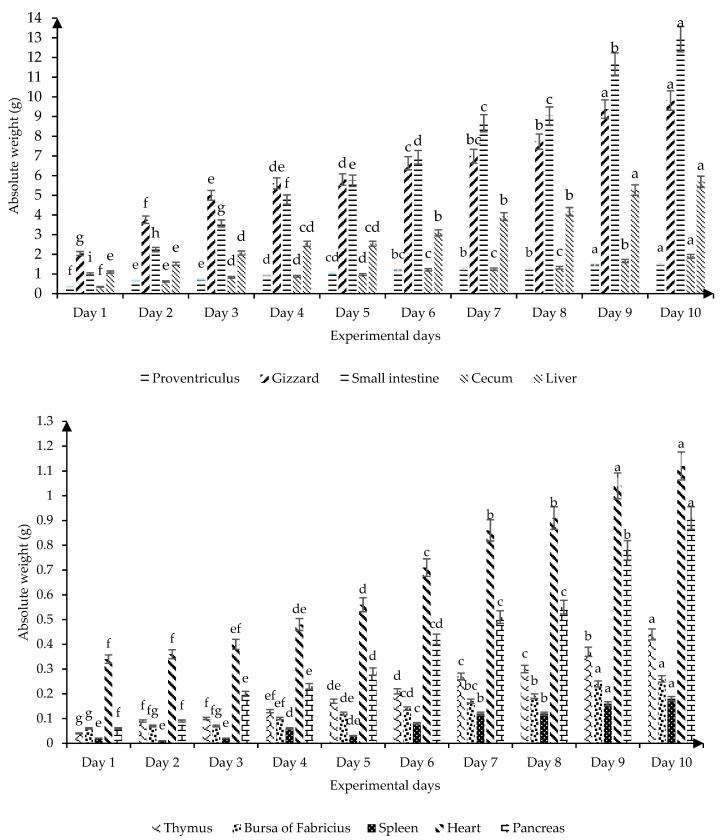
Effect of age on the absolute weight of the digestive and immune organs of broilers (1 to 10 days). n = 20. Proventriculus SEM ± 0.040, *p* value 0.001; gizzard SEM ± 0.241, *p* value 0.001; small intestine SEM ± 0.383, *p* value 0.001; cecum SEM ± 0.049, *p* value 0.001; liver SEM ± 0.157, *p* value 0.001; thymus SEM ± 0.013, *p* value 0.001; bursa of Fabricius SEM ± 0.007, *p* value 0.001; spleen SEM ± 0.067, *p* value 0.001; heart SEM ± 0.293, *p* value 0.001; pancreas SEM ± 0.029, *p* value 0.001. ^abcdefghi^ Means with different superscripts between bars differ at *p* < 0.05. SEM: Standard error of the mean.

Figure 1 shows the absolute weight of the digestive and immune organs of broilers from birth to 10 days old. All the organs measured had a progressive increase in absolute weight as the experimental days progressed (*p* ≤ 0.05); apart from the spleen, which on day 5 decreased (*p* ≤ 0.05) its absolute weight without significant differences with the first three days of the broiler’s life, while on day 10 it showed the highest absolute weight (*p* ≤ 0.05). On days 9 and 10 of life, the highest growth rate of the absolute weight of the proventriculus, gizzard, small intestine, liver, bursa of Fabricius, spleen, and heart are observed.

The effect of age on the relative weight of the digestive organs (proventriculus, gizzard, small intestine, and cecum) is shown in Table 2. The proventriculus and gizzard on the first day old have a low relative weight, however, there is an increase in the relative weight of these organs from the second to the seventh day (Table 2). On the other hand, the relative weight of the small intestine was doubled (*p* ≤ 0.05) on day two of life, and a higher weight was achieved on day nine (*p* ≤ 0.05). Similarly, the cecum increases the relative weight from the second day of life, with significant variability according to the experimental days, the third day being the most representative (*p* ≤ 0.05) (Table 2).

Age influenced the relative weight (%) of digestive viscera in the first 10 days old (Table 3). It was observed that the liver increases its relative weight from the second day of life (*p* ≤ 0.05), with the highest relative weight on the seventh day (Table 3). Also, the pancreas increased its relative weight from the third day of age and at nine days old it reached the highest relative weight (*p* ≤ 0.05). On the sixth day of life, the heart relative weight was different from the first day of life, however, the lowest value of this organ was found at 10 days old (*p* ≤ 0.05).

Thymus relative weight increased (*p* ≤ 0.05) from the second day of age and obtained its highest value on the ninth day of age, as was reported in Table 4. The bursa of Fabricius from the second day of age significantly increased (*p* ≤ 0.05) its relative weight and kept it constant without varying from the fifth day of age. The relative weight values of the spleen were low and became higher from the sixth day of age, which remained constant until the end of the experiment at 10 days old.

Table 5 shows the influence of age on lactic acid bacteria in chickens. The total lactic acid bacteria found during the 10 experimental days indicated significant differences (*p* ≤ 0.05), and the predominant lactic acid bacteria were Gram-positive rods (Table 5). Five different morphologies of colonies were identified on MRS+ MB agar, all of them Gram-positive rods: green colonies, Green colonies with white halo, white colonies, white colonies with light green halo, and flat green colonies with irregular borders. Gram-positive cocci were identified only on the first day of life. Morphological diversity of lactic acid bacteria changes during the first days of growth.

**Figure 2:**
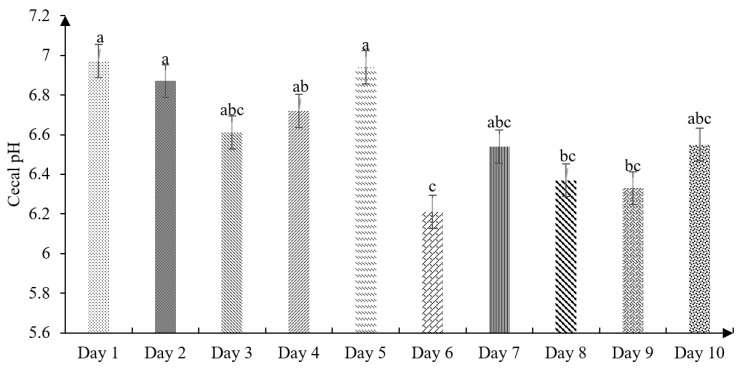
Effect of age on cecal pH in broilers (1–10 days) (SEM ± 0.146; *p* value 0.004). n = 20. ^abc^ Means with different superscripts between bars differ at *p* < 0.05. Each different bar corresponds to the cecal pH considering the experimental days.

The effect of age on the cecal pH of chickens is shown in Figure 2. The present findings showed slight cecal acidification at birth (6.97), then variable data were found up to 10 days old, showing the most acidic pH on the sixth day of life (6.21). 

In young broilers, a correlation between immune and visceral organs was found in young birds (Table 6). In this sense, the proventriculus, gizzard, small intestine, and cecum are positively correlated (*p* ≤ 0.05). Also, a significant correlation was found (Table 6) for the primary lymphoid organs (thymus and bursa of Fabricius). Likewise, the liver is correlated (*p* ≤ 0.05) with the digestive organs and with the pancreas and heart. Furthermore, the pancreas had a positive association (*p* ≤ 0.05) with the intestine, thymus, and spleen, and the heart with the spleen, liver, and pancreas (*p* ≤ 0.05).

Table 7 indicates the correlation of the relative weight of the cecum, cecal lactic acid bacteria, and the cecal pH of broilers (1–10 days). The relative weight of the cecum correlated with cecal LABs, but not for the cecal pH, which results in no association with the cecal LABs. 

## 4. Discussion

The yolk sac is the main source of energy and protein during the first days of life of the chicks [13]. Jamroz et al. [14] found an intensive absorption of the yolk sac ingredients during the first 5 days of the broiler’s life, after hatching. Although on days 7 and 16, residues of the yolk sac were found in 30 and 10% of the chickens, respectively. Thus, the broilers must have early access to the feed because it directly influences the digestive organs including the proventriculus, gizzard, and small intestine weight that increase rapidly in connection to the absolute weight of other organs and tissues [15]. Our results indicate (Figure 1) that the proventriculus and gizzard progressively increase their absolute weight with age and both organs have a similar growth rate. This result is related to the fact that these organs are stomach compartments that are physiologically connected and considered an integral part of gastric digestion [16,17]. In this sense, the proventriculus is a glandular organ (production of hydrochloric acid and pepsinogen) that transports the food bolus to the gizzard, and this organ (gizzard) due to its wide muscular layer, grinds, pulverizes, and compresses the food bolus to transport it to the intestine [18].

Also, the variability of the digestive organs in apparently healthy birds could be associated with the diet’s chemical composition, quantity, and feed form. In this sense, Huang et al. [19] found variability in the absolute weight of gizzard and cecum of broilers when they used coarse, fine, mash, and pellet diets. On the other hand, our results showed that the liver, which is the largest gland in the endocrine system, increased its absolute weight by 4.59 g in the first 10 days of life (Figure 1). This organ (liver) participates in the metabolism of proteins, carbohydrates, and lipids [20], thus an increase of the absolute liver weight in apparently healthy young birds has been related to a higher functional activity of the organ, which is essential for the assimilation of nutrients in the early stages [19,20]. Likewise, due to its exocrine and endocrine function [20], the pancreas increases its absolute weight (0.85 g; Figure 1) according to the age of the chickens, with the highest absolute values on days 9 and 10 of life. These results contribute to understanding irregularities in the absolute weight and possible activity of the digestive tract by observing drastic changes, confirming that the first 10 days are the most critical of the bird.

It is known that the study of the allometric variations of lymphoid organs could be the reflection of the immunological conditions of birds [21]. The thymus, bursa of Fabricius, and spleen showed an average absolute weight of 0.04, 0.06, and 0.04 g on the first day of life, respectively (Figure 1). Perozo et al. [21], who determined the absolute weight of the immune organs in broilers on the first day of life showed contrary results for the absolute weight of the thymus (0.15 g), however, they indicated similar results for the absolute weight of bursa of Fabricius (0.06 g) and spleen (0.03 g). It is necessary to point out that the spleen as a secondary lymphoid organ had the greatest variation in absolute weight (Figure 1). This hematopoietic organ (spleen) participates in humoral and cellular immune responses through its role in the generation, maturation, and storage of lymphocytes [21]. Ohtsu et al. [22] have mentioned that the functional activity of this organ is highly related to the genetic background of broiler hybrids, therefore, high variability would be expected, since these broilers come from different progenitors. In addition, some bacterial and viral diseases affect the functionality, morphology, and absolute weight of the spleen in broilers [21]. It is important to highlight that all the broilers remained apparently healthy during the experiment and did not have symptoms associated with any disease. 

On the other hand, the results presented on the absolute weight of the small intestine (Table 2) coincide with the reports of Noy and Uni [23] and Sklan [24], who had mentioned that the small intestine doubles its relative weight in the first 48 h of life with access to feed. These agreed with Schmidt et al. [2] who found that the small intestine of the Ross^®^ 708 genetic line has a positive allometric growth in the first few days, also, these authors [2] reported that negative allometry was observed in the small intestine on day seven. Similarly, Jaramillo [25] reported that the relative weight of the small intestine decreases on day seven because it is an organ of supply and prioritizes the development of other organs and muscles. These last reports do not coincide with the present findings (Table 2), since the relative weight of the small intestine had a proportional growth with age. Perhaps, the higher nutrient uptake reported [10] in modern fast-growing broilers is due to the positive allometry of this organ in the first 10 days, being the most critical productive stage for the absorption of nutrients. In this study, the relative weight of the cecum showed notable differences (*p* ≤ 0.05) from the second day of life (Table 2), a higher activity and growth of this organ is related to the type of diet, the amount of fermentable material, and the permanence of the feed chyme in this intestinal section [19,26].

Studies by Mateos et al. [27] found a relative liver weight of 2.55, 4.14, 3.84, and 3.09% on days 1, 4, 8, and 21 days, respectively, where the fourth day is the fastest-growing related to body weight, being different to the results shown in Table 3. These results show the genetic changes for the accelerated growth of current broilers directly influence the activity of this functional organ and its growth. In addition, Schmidt et al. [2] determined that the liver of the modern line of Ross^®^ 708 chickens grew 1,300 mg/day compared to a non-selected heritage genetic line since the 1950s. Also, as shown in Table 3, although the relative weight of the heart showed statistical differences (*p* ≤ 0.05) between the experimental days, it was found that it is the organ with the smallest growth since the first day of life (*p* ≤ 0.05). In this sense, Schmidt et al. [2] reported that the heart of the Ross^®^ 708 birds increased by 316 mg/day on average, with progressive growth until day 14. Our results showed that the relative heart weight of broilers was similar at birth and 10 days of age (Table 3). Although the results are not conclusive, the low heart growth could be associated with sudden death and wooden breast syndrome in these fast-growing broilers [28]. However, other studies are necessary to confirm this hypothesis.

The pancreas increased progressively relative weight until the ninth day old (*p* ≤ 0.05) (Table 3).

Thus, this organ in the first days is functionally immature and the digestibility of lipids, proteins, and starches is incomplete in the gastrointestinal tract [29]. In this sense, Stringhini et al. [30] evaluated the biometric of digestive organs in chickens and showed that the growth of the pancreas reached its maximum relative weight on the seventh day. Noy and Uni [23] showed that the pancreas increases its size and secretions from the fourth day due to feed intake. Our results do not coincide with this study where the relative weight of the pancreas increased concerning body weight from the third day (*p* ≤ 0.05), apparently due to the remains of the alimentary chyme in the gastrointestinal tract and the secretion of pancreatic juices to buffer the pH of the acidic chyme, which prevents damage to the small intestine.

According to Perozo et al. [21], there is a correlation between body weight and the relative weight of the lymphoid organs of broilers. The main function of the bursa of Fabricius is the maturation and differentiation of B lymphocytes that have specific immunological memory [31]. In this sense, bursa of Fabricius increased its relative weight on the second experimental day compared to the first day of life (*p* ≤ 0.05; Table 4), because this primary lymphoid organ grows directly proportional to the body weight and the immunological activity of the broilers. Likewise, Cazaban et al. [32] reported that the bursa of Fabricius uniformly increases in size as the broilers (Cobb^®^ 500 genetic lines) grow on an average absolute weight of 40 and 240 mg on days one and seven, respectively. However, Figure 1 shows that for this age in Ross 308 broilers the absolute weight of this immune organ (Bursa of Fabricius) was 60 and 170 mg, respectively.

On the other hand, Table 4 showed that the thymus on the second day doubles the relative weight (*p* ≤ 0.05), after a slight growth until the ninth day, where the highest relative weight (%) is obtained, this result is relevant considering that this organ is responsible for the differentiation and development of T lymphocytes [33], which is considered an indicator of the bird’s health status because it acts in situations of chronic stress [21]. The relative weight of the spleen did not increase until the fifth day of life (*p* ≤ 0.05), this could be attributed to the fact that, as a secondary lymphoid organ, its immunological functionality is mediated by pathogens and blood-borne antigens [22], although since on the sixth day the weight of the first days doubled without significant differences (*p* > 0.05) until the tenth day of life.

It is clear that the duodenum has the lowest population of lactic acid bacteria, and the cecum possesses the highest number of bacteria; colonization of the gastrointestinal tract occurs immediately after hatching that is influenced by the health of the environment [34]. The microflora of the intestine of birds has an essential role in digestion, metabolism, pathogen exclusion, immune stimulation, and vitamin synthesis [6]. It is necessary to consider that a larger population of lactic acid bacteria increases intestinal competitive exclusion, which is important to compete against pathogenic bacterial groups in defense of the host [35]. The composition of the bacterial community in chickens is affected by the type of bacteria that first reaches the virgin intestine [36]. Lu et al. [37] informed that the cecum is colonized mainly by anaerobic bacteria and a small number of facultative anaerobes. According to Qu et al. [38], there are a large number of unclassified cecal bacteria, which reach up to 10% of all bacteria. In this study, a similar population of total bacteria was established in the first two days of life (Table 5). Furthermore, the bacterial population did not change statistically (*p* > 0.05) from the third to the tenth day, with the fifth (8.56 log 10 CFU/g) and ninth day (8.79 log 10 CFU/g) being the ones that showed the highest count of total lactic acid bacteria. However, Saengkerdsub et al. [39] mention that the largest bacteria population is established in the first five days. These results indicate that the nutritional contributions of the diet provided directly influence the stability of the cecal microflora, essential for intestinal health and the productivity of the bird.

Diets have a significant role in cecal microbial variation, which causes changes or alterations in the cecum within 24 h after feeding the feed [40]. In a study by Ciurescu et al. [41], who measured the passage speed and intestinal pH, reported a cecal pH of 6.72 in the first week of the chicks’ life, being higher than the values found in our study (6.54; Figure 2). Therefore, Jaramillo [25] ensures that the pH variations in the cecum can be related to the type of volatile fatty acids of the diet. In this sense, Angel et al. [42] reported a pH of 6.61 on the fifth day of life, which is different from the results shown in this study (6.94). As well, Huang et al. [19] found a cecal pH of 6.01 when using a fine particle diet. 

On the other hand, the decrease in intestinal pH favors the growth of beneficial bacteria, such as *Lactobacillus* spp. and *Bifidobacterium* spp., and reduces the growth of pathogenic bacteria, such as *E. coli* and *Salmonella* spp. through competitive intestinal exclusion. Furthermore, homo and hetero-fermentative lactic acid bacteria produce bacteriocin’s that prevent colonization by undesirable, non-proliferating bacteria [43]. Likewise, Molina et al. [12] and Lópes et al. [44] found that the use of *Ganoderma lucidum* and yeasts as functional additives in broilers caused a cecal pH of 6.45 and 6.0 on the tenth and eighth day of life, respectively, being differences to our findings. The changes in cecal pH will depend on the growth of cecal lactic acid bacteria that decrease the cecal pH due to the production of volatile fatty acids [45], although you also have to consider the feed presentation form and the use of growth-promoting antibiotics and natural alternatives such as probiotics, prebiotics, and phytobiotics. It should be noted that this study considered diets in the mash form and free of zootechnical additives (including antibiotic growth promoters).

The proventriculus, gizzard, small intestine, and cecum are also associated (*p* ≤ 0.05) with the liver and pancreas, because higher feed intake increases hepatic lipid metabolism and the production of pancreatic enzymes, such as HMG-CoA reductase, trypsinogen, chymotrypsinogen, lipase, amylase, ribonucleases, carboxypeptidase, and deoxyribonuclease that are secreted and discharged through the pancreatic ducts and common bile duct [46]. 

The liver is positively correlated (*p* ≤ 0.05) with the small intestine because this viscera contributes to the production of IgA by the immune system, and IgA is responsible for protecting the walls of the intestine and preventing the adherence of pathogens to the intestinal mucosa [47]. Also, the pancreas and liver are positively correlated by homeostatic regulation for the segregation of insulin and glucagon [48], which helps maintain stable glucose levels. In addition, the small intestine is correlated with the lymphoid organs (thymus, spleen, and bursa of Fabricius) because higher activity and intestinal health ensures higher production of T and B lymphocytes and increases the relative weight of these hematopoietic organs in the young bird [49,50]. It should also be noted that the intestine has a unique enteric nervous system and concentrates 70% of the immune cells [51], which means that increased absorption of nutrients is essential for the immune system and the future development of the broiler.

Tambini et al. [52] found a positive correlation between the bursa of Fabricius and the spleen in broilers raised for 49 days. However, the present findings shown in Table 6 indicated that these immune organs (bursa of Fabricius and the spleen) were not significantly correlated in young birds (up to 10 days). In this sense, the bursa of Fabricius has been reported to grow at a circadian rate higher than the spleen in the early days of birds [53]; (Figure 1), since the greatest activity of the spleen as a secondary lymphoid organ occurs in adult birds. However, Perozo et al. [21] reported a correlated association between thymus and spleen; similar results are seen in Table 6. Likewise, the liver, spleen, and heart have a positive correlation due to hepatic portal blood circulation; the venous blood that arises from the gastrointestinal organs and the spleen is rich in the digestive substance’s tract [53,54]. These results coincide with Philipsen et al. [55], who showed a positive correlation between the liver and heart, as they connected through the portal vein, which contributes to the detoxification and exchange of arterial blood. 

García et al. [56] indicate that a greater presence of cecal lactic acid bacteria (LAB) decreases the pH. It is important to note that despite a greater beneficial bacterial population as the bird grows, this was not enough to correlate it with cecal pH. Martínez et al. [57] found that the use of a natural product rich in beneficial secondary metabolites in the diets of young birds did not statistically vary the cecal pH, perhaps due to the late proliferation of cecal lactic acid bacteria (*p* ≤ 0.05) at early ages.

Furthermore, in Table 7 a positive correlation was observed between the relative weight of the cecum and the cecal lactic acid bacteria count (*p* ≤ 0.05); apparently, they are biologically associated, since the increase in cecal bacterial proliferation causes a greater fermentative activity in this organ [54], although influenced by the age of the birds (Table 2). In this sense, Latorre et al. [58] reported that higher colonization of bacteria in the cecum induces eminent functioning of the organ, which grows its relative weight due to the more presence of produced volatile fatty acids. 

## 5. Conclusions

The results showed that the absolute weight of the immune organs and viscera increases with age, with greater emphasis on days 9 and 10. Contrary to the relative weight of the organs that varied on the experimental days. Cecal lactic acid bacteria are established before 10 days of life and a high variability in intestinal pH was found. The correlation analysis showed a significant association between all organs measured, as well as for the relative weight and the cecal lactic bacteria count. Therefore, these results could contribute to the understanding of the gut physiology and microbiology of the first days of life of modern broilers, which is crucial to improve the genetic expression of these animals.

## Figures and Tables

**Table 1:** Ingredients and contributions of broiler diets (1–10 days).

Ingredients	Percentages (%)
Cornmeal (crude protein, 8.3%)	49.79
Soymeal (crude protein, 48%)	39.54
Mineral and vitamin premix ^1^	0.50
Sodium chloride	0.50
African palm oil	6.15
Choline	0.08
DL-Methionine	0.38
L-Threonine	0.10
L-Lysine	0.25
Calcium carbonate	1.13
Monocalcium phosphate	1.58
Proximal composition (%)	
Metabolizable energy (kcal/kg dry matter)	3000
Crude protein	23.43
Crude fiber	2.39
Ashes	6.36
Ca	0.96
*p* available	0.48
Methionine + Cystine	0.95
Threonine	0.86
Valine	0.91
Isoleucine	0.80
Leucine	1.60
Lysine	1.28
Histidine	0.51
Arginine	1.30
Tryptophan	0.24
Phenylalanine	0.80

^1^ Each kg contains: vitamin A, 13,500 UI; vitamin D3, 3375 UI; vitamin E, 34 mg; B2, 6 mg; pantothenic acid, 16 mg; nicotinic acid, 56 mg; Cu, 2000 mg; folic acid, 1.13 mg; vitamin B12, 34 mg; Mn, 72 mg; Zn, 48 mg.

**Table 2:** Effect of age on the relative weight of the digestive organs of broilers (1–10 days).

Days	Digestive Organs (%)			
	Proventriculus	Gizzard	Small Intestine	Cecum
1	0.78 ^e^	4.32 ^e^	2.15 ^f^	0.73 ^d^
2	1.39 ^ab^	7.66 ^b^	4.58 ^e^	1.27 ^abc^
3	1.32 ^abc^	8.95 ^a^	6.39 ^d^	1.49 ^a^
4	1.47 ^a^	8.95 ^a^	7.39 ^c^	1.36 ^abc^
5	1.42 ^a^	7.58 ^b^	7.78 ^bc^	1.30 ^abc^
6	1.40 ^ab^	7.63 ^b^	7.95 ^bc^	1.39 ^ab^
7	1.24 ^bc^	7.45 ^bc^	8.33 ^b^	1.19 ^bc^
8	1.18 ^cd^	6.29 ^d^	8.20 ^b^	1.19 ^bc^
9	1.03 ^d^	6.91 ^cd^	9.08 ^a^	1.35 ^abc^
10	1.06 ^d^	6.34 ^d^	7.92 ^bc^	1.14 ^c^
SEM±	0.055	0.217	0.239	0.074
*p* value	<0.001	<0.001	<0.001	<0.001

^abcdef^ Means with different letters in the same column differ from *p* ≤ 0.05. n = 20. SEM: Standard error of the mean.

**Table 3:** Effect of age on the relative weight of some viscera of broilers (1–10 days).

Days	Digestive Viscera (%)		
	Liver	Pancreas	Heart
1	2.33 ^e^	0.12 ^d^	0.72 ^bc^
2	3.10 ^d^	0.19 ^d^	0.73 ^bc^
3	3.72 ^abc^	0.36 ^c^	0.72 ^bc^
4	3.91 ^ab^	0.35 ^c^	0.74 ^abc^
5	3.45 ^cd^	0.37 ^c^	0.75 ^abc^
6	3.56 ^bc^	0.48 ^b^	0.82 ^ab^
7	4.02 ^a^	0.49 ^b^	0.83 ^a^
8	3.54 ^bc^	0.49 ^b^	0.82 ^ab^
9	3.72 ^abc^	0.64 ^a^	0.79 ^abc^
10	3.82 ^abc^	0.53 ^b^	0.71 ^c^
SEM±	0.142	0.031	0.031
*p* value	<0.001	<0.001	0.017

^abcde^ Means with different letters in the same column differ from *p* ≤ 0.05. n = 20. SEM: Standard error of the mean.

**Table 4:** Effect of age on the relative weight of the immune organs of broilers (1–10 days).

Days	Immune Organs (%)		
	Thymus	Bursa of Fabricius	Spleen
1	0.09 ^e^	0.12 ^b^	0.035 ^c^
2	0.18 ^d^	0.16 ^a^	0.028 ^c^
3	0.18 ^d^	0.12 ^b^	0.026 ^c^
4	0.20 ^cd^	0.15 ^ab^	0.086 ^b^
5	0.22 ^bcd^	0.16 ^a^	0.038 ^c^
6	0.24 ^bc^	0.16 ^a^	0.094 ^ab^
7	0.26 ^b^	0.17 ^a^	0.113 ^ab^
8	0.27 ^b^	0.17 ^a^	0.111 ^ab^
9	0.31 ^a^	0.17 ^a^	0.124 ^a^
10	0.25 ^b^	0.18 ^a^	0.109 ^ab^
SEM±	0.016	0.010	0.011
*p* value	<0.001	0.003	<0.001

^abcde^ Means with different letters in the same column differ from *p* ≤ 0.05. n = 20. SEM: Standard error of the mean.

**Table 5:** Effect of age on cecal lactic acid bacteria count of broilers (1–10 days).

Days	Cecal Lactic Acid Bacteria (log 10 CFU/g)						
	Bacilli^1^	Cocci^2^	Bacilli^3^	Bacilli^4^	Bacilli^5^	Bacilli^6^	Total
1	6.14 ^b^	5.53	4.00 ^b^	NP	NP	NP	6.11 ^c^
2	7.62 ^ab^	NP	4.00 ^b^	4.00^c^	NP	NP	6.63 ^bc^
3	7.16 ^ab^	NP	NP	5.00 ^bc^	4.82	NP	7.99 ^ab^
4	9.01 ^a^	NP	4.98 ^ab^	5.23 ^bc^	NP	NP	8.03 ^ab^
5	8.54 ^a^	NP	4.41 ^ab^	5.10 ^bc^	NP	NP	8.56 ^a^
6	8.68 ^a^	NP	4.48 ^ab^	NP	NP	4.00	7.69 ^abc^
7	7.94 ^ab^	NP	6.32 ^a^	5.06 ^bc^	NP	5.13	8.04 ^ab^
8	8.24 ^a^	NP	5.20 ^ab^	6.99 ^ab^	NP	NP	8.38 ^ab^
9	8.78 ^a^	NP	4.00 ^b^	6.02 ^abc^	NP	NP	8.79 ^a^
10	8.18 ^ab^	NP	4.00 ^b^	7.85 ^a^	NP	NP	8.35 ^ab^
SEM±	0.639		0.619	0.727		0.797	0.539
*p* value	0.019		0.018	0.037		0.394	0.041

^abc^ Means with different letters in the same column differ from *p* ≤ 0.05. n = 5. Colony and microscopic morphology on MRS+MB agar: Bacilli^1^ = Green colonies Gram-positive rods; Cocci^2^ = White colonies, Gram-positive cocci; Bacilli^3^ = Green colonies with white halo Gram-positive rods; Bacilli^4^ = White colonies Gram-positive rods; Bacilli^5^ = Green colonies with light green halo Gram-positive rods; Bacilli^6^ = Irregular flat green colonies Gram-positive rods. NP = no presence. SEM: Standard error of the mean.

**Table 6:** Pearson’s correlation between the digestive and immune organs of broilers.

	Gizzard	Small Intestine	Cecum	Thymus	Spleen	Liver	Pancreas	Heart	Bursa of Fabricius
Proventriculus	0.603 **	0.330 **	0.399 **	0.133	–0.108	0.217 *	0.070	–0.016	0.074
Gizzard		0.394 **	0.595 **	0.168	–0.088	0.437 **	0.152	0.018	0.079
Small intestine			0.407 **	0.674 **	0.520 **	0.570 **	0.719 **	0.175	0.297 **
Cecum				0.275 **	–0.020	0.355 **	0.294 **	0.051	0.145
Thymus					0.444 **	0.498 **	0.676 **	0.164	0.343 **
Spleen						0.530 **	0.617 **	0.29 **	0.174
Liver							0.572 **	0.302 **	0.087
Pancreas								0.254 *	0.260 **
Heart									0.121

** The correlation is significant at *p* ≤ 0.01; * The correlation is significant at *p* ≤ 0.05. n = 20.

**Table 7:** Pearson’s correlation between the relative weight of the cecum, cecal lactic acid bacteria count, and the cecal pH of broilers.

Items	Cecal pH	Lactic Acid Bacteria (Log 10 CFU/g)
Cecum (g)	–0.254	0.561 **
Cecal pH		–0.242

** The correlation is significant at *p* ≤ 0.05. n = 20.

## Data Availability

Not Applicable.
